# Vitamin D Supplementation and Cardiovascular Disease Risks in More Than 134000 Individuals in 29 Randomized Clinical Trials and 157000 Individuals in 30 Prospective Cohort Studies: An Updated Systematic Review and Meta-analysis

**DOI:** 10.34172/jrhs.2023.129

**Published:** 2023-12-29

**Authors:** Mohammad Aziz Rasouli, Shahram Darvishzadehdaledari, Zeynab Alizadeh, Ghobad Moradi, Fatemeh Gholami, Ako Mahmoudian

**Affiliations:** ^1^Department of Epidemiology and Biostatistics, Faculty of Medicine, Kurdistan University of Medical Sciences, Sanandaj, Iran; ^2^Social Determinants of Health Research Center, Research Institute for Health Development, Kurdistan University of Medical Sciences, Sanandaj, Iran; ^3^Department of Health Sciences, University of York, Heslington, York, YO 10 DD, United Kingdom; ^4^Department of Epidemiology, School of Public Health, Iran University of Medical Sciences, Tehran, Iran; ^5^Rajaie Cardiovascular Medical and Research Center, Iran University of Medical Sciences, Tehran, Iran

**Keywords:** Vitamin D, Cardiovascular disease, Randomized clinical trials, Prospective cohort study, Meta-analysis

## Abstract

**Background:** According to the findings from observational studies and clinical trials assessing the effect of vitamin D supplements on cardiovascular diseases (CVDs), there are still contradictory results. This systematic review aimed to assess the effect of vitamin D supplements on CVDs considering cohort studies and clinical trials.

**Study Design:** A systematic review.

**Methods:** MEDLINE/PubMed, Science Direct, Embase, and Cochrane Library databases were reviewed by two reviewers independently until 2022. The study effect is risk ratio (RR) and 95% confidence interval (CI) according to Mantel Haenszel’s random-effects model. Then, Stata version 14 was used for statistical analysis.

**Results:** In clinical trial studies, the incidence of CVDs among the vitamin D-consuming group was not significantly different from that in the placebo group (RR: 0.99, 95% CI: 0.95-1.03; *P*=0.77; I ^2^=0%). CVD mortality was also not significantly different between the two groups (RR: 0.97, 95% CI: 0.90-1.05; *P*=0.72; I^2^=0%). In cohort studies, circulating 25 (OH) D increased the risk of CVD incidence by 31% (RR: 1.31, 95% CI: 1.19-1.45) and CVD mortality by 37% (RR: 1.37, 95% CI: 1.17-1.61).

**Conclusion:** According to current evidence from clinical trials, vitamin D supplementation should not be recommended for CVD prevention. However, there is a direct association between vitamin D deficiency and the incidence of CVDs as well as its mortality. According to the results of clinical trial studies carrying higher levels of scientific evidence, it can be concluded that vitamin D supplementation does not exert a significant effect on the incidence, mortality, and reduction of CVDs.

## Background

 Today, despite significant progress in access to effective and safe prevention strategies all around the world, cardiovascular diseases (CVDs) still tend to remain one of the major causes of death.^1,2^ The prevalence of CVDs is increasing in developed and developing countries, where it imposes a heavy financial burden on different populations.^3,4^ In addition to traditional and recognized risk factors for CVDs, new risk factors are potentially associated with prognosis and therapeutic consequences.^5^ The most common risk factors associated with CVDs are predominantly obesity, diabetes, high blood pressure, and inactivity.^2,6^ Nevertheless, the results of numerous studies illustrated that insufficient levels or the lack of vitamin D may increase the risk of CVDs.^7,8^

 Numerous factors influence vitamin D deficiency, including older women living in places with higher latitude, winter season, less exposure to sunlight, skin pigmentation, diet, and the consumption of fortified foods with low levels of vitamin D.^9^ In all age groups, the prevalence of vitamin D deficiency has been estimated to be 30-50%.^10^ A study was designed to determine the vitamin D status of 60 979 patients admitted to the Burjeel hospital of VPS Healthcare in Abu Dhabi, United Arab Emirates (UAE), from October 2012 to September 2014. Although analyzed patients were from 136 different countries, serum 25(OH)D (total) measurements showed that 82.5% of the studied patients have vitamin D deficiency to insufficiency.^11^

 The low rates of vitamin D are associated not only with CVD risk but also with deterioration of current cardiac status. The results of several observational epidemiologic studies have shown that a lack of vitamin D efficiency increases the probability of myocardial infarction (MI), stroke, heart attack, and CVDs-related mortality.^12,13^ Although clinical data provide several beneficial effects of vitamin D on CVDs, at best conditions, elevated doses of vitamin D can cause moderate impacts on alternative parameters of CVDs, according to findings from Genetic studies and clinical trials.^14,15^ Generally, vitamin D deficiency can develop short-term and long-term prognoses for CVDs.^16,17^

 According to the findings from observational studies and clinical trials assessing the effect of vitamin D supplements on CVDs, there are still contradictory results.^17-21^ Although meta-analysis studies have been conducted, cohort articles and clinical trials have not been considered together. Accordingly, this systematic review and meta-analysis aimed to update the effect of vitamin D supplements on CVDs considering cohort studies and clinical trials.

## Methods

###  Search strategy 

 This systematic review was conducted based on the Preferred Reporting Items for Systematic Reviews and Meta-Analyses (PRISMA) standard checklist.^22^ The study protocol was registered in the International Prospective Register of Systematic Reviews (PROSPERO identifier: CRD42022360801). MEDLINE/PubMed, Science Direct, Embase, and Cochrane Library databases were reviewed by two reviewers independently until October 2022. There were no age or gender restrictions, and all available references regarding systematic reviews and meta-analyses were evaluated.

###  PICOS criteria

*Population*: Populations without CVDs

*Intervention*: Vitamin D supplements

*Control*: Placebo, and Placebo + Calcium

*Outcome*: CVD, chronic heart failure (HF), MI, and stroke

*Studies*: Randomized controlled trials (RCTs) and prospective cohort studies (PCSs)

###  Selection criteria 

 This study included all prospective cohorts and clinical trials evaluating long-term (more than one year) vitamin D intake with or without calcium which were assessed based on our intended outcomes. The search strategy was vitamin D3 OR cholecalciferol OR ergocalciferol, OR 25 (OH) D AND cardiovascular OR chronic heart failure OR myocardial infarction OR stroke OR cerebrovascular OR chronic heart disease AND randomized controlled trials OR randomized trials OR controlled trials OR prospective cohort OR cohort studies.

 Studies investigating CVDs as adverse events were included in this research. HF disease was classified under chronic HF. Cerebrovascular disease was considered a subset of stroke. In clinical trials, ischemic heart disease was included in the MI subcategory. Studies excluded from this review included nested case-control studies, cross-sectional papers, case-control studies, case studies, case reports, poster abstracts, editorials, trials identifying their control group as non-placebo, populations receiving different doses of vitamin D, trials recruiting pregnant and lactating women, trials in which all study subjects suffered from CVDs, trials in which the comparison group only received calcium, and studies not evaluating our intended outcome.

###  Data extraction and quality assessment 

 Two reviewers (FG and SD) extracted the relevant data independently in a specified data collection table. Any discrepancies between reviewers were resolved by a third author (MAR) Different variables, including the type of study, country, gender, mean age, follow-up period, and quality of the study were assessed. The inter-authors’ reliability based on kappa statistics was 85%.

 Newcastle Ottawa and risk of bias (ROB2) tools were used for cohort studies and clinical trials, respectively, aiming at assessing the quality of papers. In Newcastle Ottawa, studies obtaining nine stars were classified as high quality, those with 7 or 8 stars were entitled as moderate, and papers with six stars or below were grouped in the low-quality category.^23,24^ In ROB2, five domains encompassing the randomization process, deviations from intended interventions, missing outcome data, measurement of the outcome, and selection of the reported result were evaluated ^25^.

###  Outcomes and subgroups 

 In this study, the primary endpoint was CVD events and deaths, and the secondary endpoints were MI, stroke, and chronic heart disease (CHD). In some studies, the follow-up period for different outcomes is variable; thus, a specific follow-up period is given for each outcome.

###  Statistical analysis

 The study effect is risk ratio (RR) and 95% confidence intervals (CIs) according to Mantel Haenszel’s random-effects model. Publication bias was evaluated by using a funnel plot^26^ and Egger’s test,^27^ and I^2^ based on Higgins classification^28^ was used to measure heterogeneity. The sensitivity analysis of the primary endpoint was measured by excluding each study.^29^ Furthermore, subgroup analysis was performed in accordance with the study covariates such as gender, duration of follow-up, and the quality of study. Moreover, in PCSs, in addition to the above factors, baseline CVD history was also used. To avoid spurious inferences from repeated significant tests and underpowered meta-analysis, we performed a sequential trial analysis. We were able to obtain reliable results using sequential monitoring boundaries.^30^ We calculated the optimal information (sample) size by considering 2-sided type I error at 5% level and type II error at 20% level (80% power), with a relative risk reduction of 25% and incidence of 8.5% in the placebo group for the CVDs incidence. Finally, STATA version 14 (Stata Corporation, Texas, USA) was used for statistical analysis.

## Results

###  Study selection and study characteristics 

**Figure 1:**
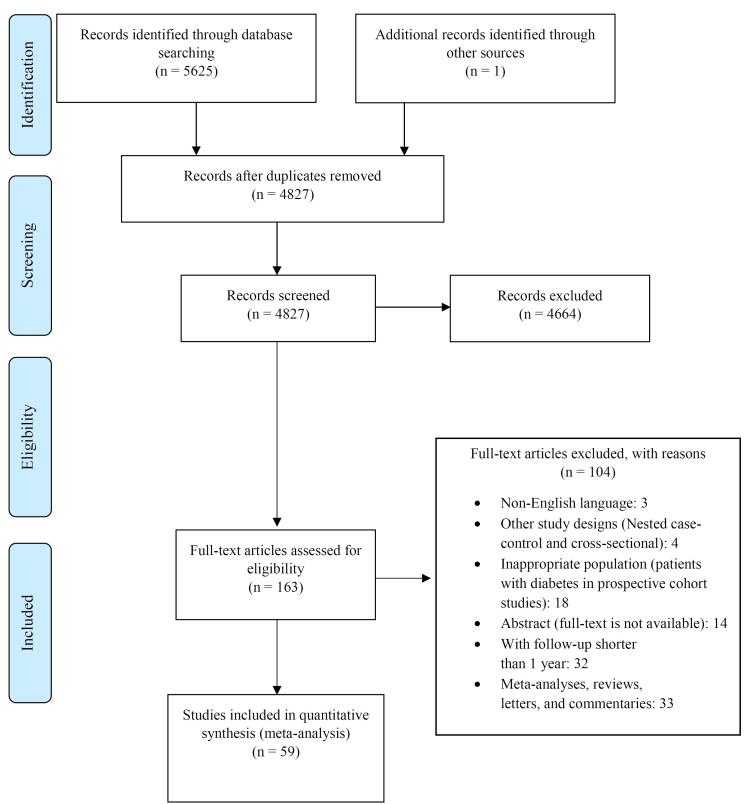
Flow diagram of the study selection process

 After reviewing 5626 studies from the databases, 4664 papers were excluded, but 29 RCT and 30 PCSs were selected for the final analysis. Figure 1 illustrates the process of selecting studies. Among 134 384 participants entering the clinical trials, 67 665 were taking vitamin D, and 66 719 were not taking vitamin D. In clinical trials, 17 studies (58.6%) were classified as low risk, four studies (13.8%) as some concerns, and eight studies (27.6%) as high risk. As mentioned, most of the studies were in the low-risk category (Figures S1 and S2). Table 1 presents the basic characteristics of participants in RCTs. In terms of PCSs, eight studies (26%) were in the high-quality category, and 22 studies (74%) were in the moderate-quality category. Moreover, 157 958 individuals participated in the PCS, of which 70 009 were in the exposed group, and 87 949 were in the unexposed group. The baseline information of the participants in PCSs can be seen in Table 2.

**Table 1:** Characteristics of studies on vitamin d and cardiovascular diseases in RCT

**Author, Year**	**Country**	**Mean age (y)**	**Intervention**	**Control**	**Gender**	**Quality (ROB2)**	**Follow-up (y)**
Virtanen, 2022 ^31^	USA	68	D3 1600 IU/D, D3 3200 IU/D, D3 1600 + 3200 IU/D	Placebo	Both	Low risk	5
Neale,2022 ^32^	Australia	69	D3 60000 IU/M	Placebo	Both	Low risk	5.7
Chatterjee, 2021 ^33^	USA	60	D3 4000 IU/D	Placebo	Both	Low risk	2.9
Manson,2019 ^18^	USA	67.1	D3 2000 IU/D	Placebo	Both	Low risk	5.3
Shoji,2018 ^34^	Japan	65	Alfacalcidol 0.5 μg/D	Placebo	Both	Low risk	4
Scragg,2017 ^35^	New Zealand	65.5	D3 100000 IU/M	Placebo	Both	Low risk	3.3
Zittermann, 2017 ^36^	Germany	55	D3 4000 IU/D	Placebo	Both	Low risk	3
Jorde,2016 ^37^	Norway	62	D3 20,000 IU/W	Placebo	Both	Moderate	5
Baron,2015 ^38^	USA	58	Ca1200 mg + D3 1000 IU/D	Placebo + Calcium	Both	Low risk	3
Martineau,2014 ^39^	UK	67.1	D3, 3 mg (120,000 IU)/2M	Placebo	Both	Low risk	1
Ford,2014 ^40^	UK	77.5	D3 800 IU/D	Placebo	Both	Low risk	2
Wang,2014 ^41^	Hong Kong	61	Paricalcitol 1 μg/D	Placebo	Both	Moderate	1
Witham,2013 ^42^	UK	77	D3 100000 IU/3M	Placebo	Both	Low risk	1
Gallagher, 2012 ^43^	USA	67	Calcitriol, 0.25 μg twice/D	Placebo	Female	Low risk	1
Lehouck, 2012 ^44^	Belgium	60	D3 100000 IU/M	Placebo	Both	Low risk	1
Sanders,2010 ^45^	Australia	76.5	D3 500000 IU/Y	Placebo	Female	Low risk	2.96
Prince,2008 ^46^	Australia	77	Ca 1000mg + D3 1000 IU/D	Placebo + Calcium	Female	Low risk	1
Zhu,2008 ^47^	Australia	74.8	Ca 1200 mg + D3 1000 IU/D	Placebo + Calcium	Female	Moderate	5
Berggren, 2007 ^48^	Sweden	82	Ca 1000 mg + D3 800 IU/D	Placebo	Female	High risk	1
Hsia,2007 ^49^	USA	62	CaCO3 500 mg + D3 200 IU twice /D	Placebo	Female	High risk	7
Jackson,2006 ^50^	WHI, USA	62.4	D3 400 IU/D	Placebo	Female	Low risk	12
Brazier,2005 ^51^	France	76.4	CaCO3 500 mg + D3 400 IU twice /D	Placebo	Female	High risk	1
Grant,2005 ^52^	UK	77	D3 800 IU/D	Placebo	Both	Low risk	3.8
Trivedi,2003 ^53^	UK	74.8	D3 100000 IU/4M	Placebo	Both	Moderate	5
Komulainen,1999 ^54^	Finland	53	D3 100 and 300 IU/D	Placebo	Female	High risk	5
Ott,1989 ^55^	USA	67.9	D3 1000 mg/D	Placebo	Female	High risk	2
Aloia,1988 ^56^	USA	64.1	D3 400 IU/D	Placebo	Female	High risk	2
Inkovaara, 1983 ^57^	Finland	79.5	D3 1000 IU/D	Placebo	Both	High risk	1
Brohult,1973 ^58^	Sweden	52	D3 100000 IU/D	Placebo	Both	High risk	1

*Note.* RCT: Randomized controlled trial; ROB2: Risk of bias.

**Table 2:** Characteristics of studies on vitamin d and cardiovascular diseases in PCSs

**Author, Year**	**Country**	**Mean age (y)**	**Exposed (nmol/L)**	**Unexposed (nmol/L)**	**Gender**	**Quality (NOS)**	**Follow-up (y)**
Park,2022 ^21^	Korea	50	≥ 50	< 30	Both	Moderate	8.9
Heath,2020 ^59^	Australia	61.3	Female: 53.1-121.3 Male: 68.9-201.8	Female: 13.9-34.7 Male: 8.2-42.9	Both	Moderate	13.7
Paul,2019 ^60^	UK	65	> 84	≤ 41.25	Both	Moderate	4
Crowe,2019 ^61^	UK	52.1	67.50-206.49	0.05-23.09	Both	Moderate	2.2
Su,2019 ^62^	China	73	50 - < 125	< 25	Both	High	13.8
Leo Agelii,2017 ^63^	Sweden	47	> 51.45	≤ 51.45	Female	Moderate	17
El Hilali,2015 ^64^	Netherlands	75	≥ 75	< 25	Both	High	13
Lutsey,2015 ^65^	USA	56.5	87.75 (median)	35 (median)	Both	Moderate	18
Chien,2015 ^66^	China	60	≥ 63.8	< 39	Both	Moderate	9.6
Michos,2015 ^67^	USA	56	≥ 75	< 50	Both	High	19.7
Khaw,2014 ^68^	UK	63	≥ 90	< 30	Both	Moderate	13
Wannamethee,2014 ^69^	UK	68	≥ 65	< 35	Male	High	13
Perna,2013 ^70^	Germany	50	≥ 50	< 30	Both	Moderate	8
Bajaj,2013 ^71^	USA	67	≥ 50	< 50	Male	Moderate	4.4
Schöttker,2013 ^72^	Germany	62	> 50	< 30	Both	Moderate	9.5
Rohrmann, 2013 ^20^	Switzerland	47.1	62.5-249.5	0-33.5	Both	Moderate	17.6
Kühn,2013 ^73^	Germany	53	≥ 50	< 25	Both	Moderate	7.7
Robinson-Cohen,2013 ^74^	USA	61	≥ 75	< 50	Both	Moderate	8.5
Schierbeck,2012 ^75^	Denmark	50	≥ 50	< 50	Female	Moderate	16
Lin,2012^76^	USA	56	≥ 48.4	< 19.6	Both	Moderate	24
Kritchevsky,2012 ^77^	USA	74.5	≥ 75	< 25	Both	Moderate	8.5
Messenger,2012 ^78^	USA	76.5	75.5-138.5	12.25-50.25	Male	Moderate	4.4
Kestenbaum,2011 ^79^	USA	73.5	> 75	< 37.5	Both	High	14
Bansal,2014 ^80^	UK	62.1	≥ 75	< 50	Both	Moderate	8.46
Bolland,2010 ^81^	New Zealand	74	≥ 50	< 50	Female	Moderate	5
Hutchinson,2010^82^	Norway	60	72.3 (median)	33.8 (median)	Both	Moderate	11.7
Michaëlsson,2010 ^83^	Sweden	71	> 98	< 39	Male	High	12.7
Kilkkinen,2009 ^84^	Finland	49.4	Female: 56-151 Male: 62-180	Female: 4-25 Male: 5-28	Both	High	27.1
Giovannucci,2008 ^85^	USA	63.8	≥ 75	< 37.5	Male	High	10
Pilz,2008 ^86^	Germany	63	50-74.99	< 25	Both	Moderate	7.7

*Note.* PCS: Prospective cohort study; NOS: Newcastle-Ottawa scale.

###  Primary endpoint: randomized controlled trial studies

**Figure 2:**
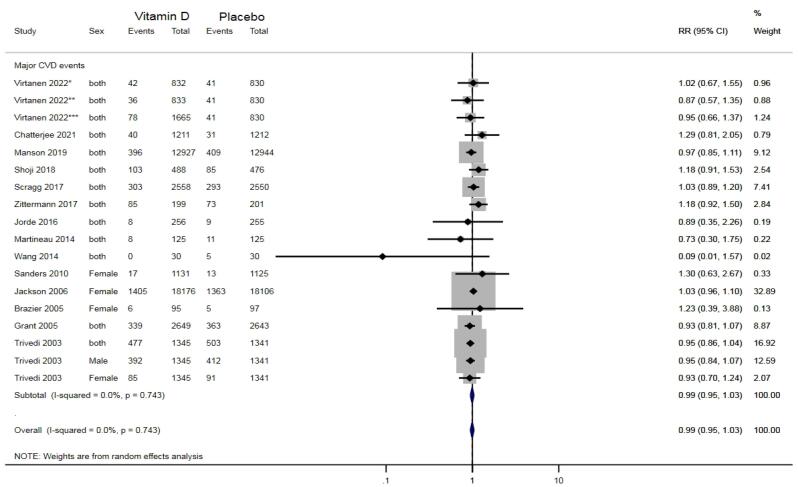
Forest plot for the results of the primary end point (cardiovascular events) in RCTs. *Note.* RCT: Randomized controlled trial; * Intervention: Vitamin D 1600 IU/Day; ** Intervention: Vitamin D 3200 IU/Day; *** Intervention: Vitamin D 1600 + 3200 IU/Day

**Figure 3:**
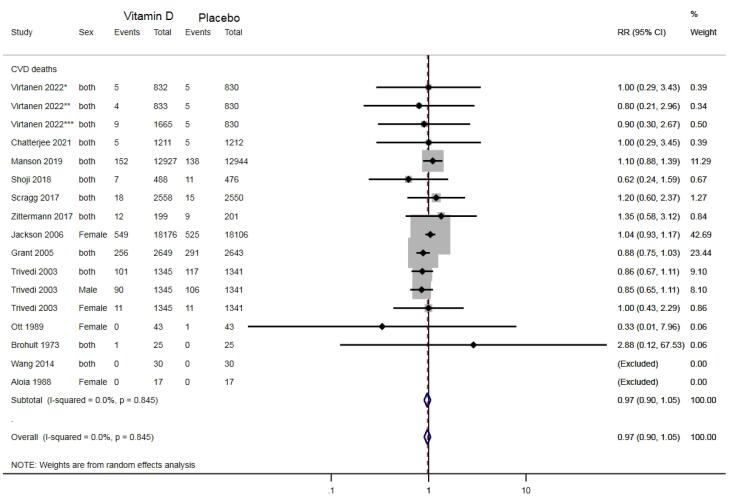
Forest plot for the results of the primary end point (cardiovascular deaths) in RCTs. *Note.* RCT: Randomized controlled trial; * Intervention: Vitamin D 1600 IU/Day; ** Intervention: Vitamin D 3200 IU/Day; *** Intervention: Vitamin D 1600 + 3200 IU/Day

 In clinical trial studies, the incidence of CVDs among the vitamin D-consuming group was not significantly different from that in the placebo group (RR: 0.99, 95% CI: 0.95-1.03; *P* = 0.77; I^2^ = 0%), as illustrated in Figure 2. As illustrated in Figure 3, CVD mortality was also not significantly different between the two groups (RR: 0.97, 95% CI: 0.90-1.05; *P* = 0.72; I^2^ = 0%).

 The *P* value of Egger’s test for the CVD events was 0.87, and CVD mortality was 0.75. Subgroup analysis was conducted for the main outcome based on vitamin D types, gender, duration of follow-up, and study quality. Table 3 depicts that the effect size was not significant in any of the studies. Moreover, the incidence of MI and stroke in the vitamin D-consuming group was not significantly different from the placebo group (RR:1.01, 95% CI: 0.95-1.07; RR: 1.04, 95% CI: 0.97-1.10), as depicted in Figures S3 and S4.

**Table 3:** Subgroup analysis in RCTs and PCSs

**Subgroup**	**Studies design**	**No. of effects**	**RR (95% CI)**	**I**^2^	***P***** value**
CVD events by gender					
Both	RCT	13	0.98 (0.93-1.04)	0%	0.573
Male	RCT	1	0.95 (0.84-1.07)	-	-
Female	RCT	4	1.02 (0.96-1.10)	0 %	0.813
CVD mortality by gender					
Both	RCT	12	0.94 (0.84-1.04)	0 %	0.852
Male	RCT	1	0.85 (0.65-1.11)	-	-
Female	RCT	4	1.04 (0.92-1.17)	0 %	0.777
CVD events by vitamin D3	RCT	15	0.99 (0.95-1.03)	0 %	0.874
CVD mortality by vitamin D3	RCT	15	0.97 (0.90-1.05)	0 %	0.851
CVD events by ROB2					
Low risk	RCT	12	1.02 (0.97-1.07)	0%	0.763
Some concerns	RCT	5	0.94 (0.88-1.02)	0%	0.621
High risk	RCT	1	1.23 (0.39-3.88)	-	-
CVD mortality by ROB2					
Low risk	RCT	10	1.00 (0.92-1.09)	0%	0.776
Some concerns	RCT	4	0.86 (0.72-1.03)	0%	0.935
High risk	RCT	3	0.99 (0.11-9.24)	0%	0.344
CVD events by follow-up (y)					
> 3	RCT	12	0.99 (0.95-1.03)	0%	0.882
≤ 3	RCT	6	1.16 (0.95-1.42)	0%	0.476
CVD mortality by follow-up (y)					
> 3	RCT	11	0.97 (0.90-1.05)	0%	0.698
≤ 3	RCT	6	1.20 (0.62-2.64)	0%	0.782
Myocardial infarction by vitamin D3	RCT	20	0.99 (0.93-1.06)	0%	0.999
Stroke by vitamin D3	RCT	18	1.04 (0.97-1.12)	0%	0.986
CVD events by gender					
Both	PCS	5	1.44 (1.27-1.63)	51.9%	0.081
Male	PCS	2	1.03 (0.88-1.22)	0%	0.498
Female	PCS	4	1.19 (0.94-1.51)	80.6%	0.001
CVD mortality by gender	PCS				
Both	PCS	12	1.40 (1.18-1.67)	72.3%	0.001
Male	PCS	1	0.90 (0.39-2.05)	-	-
Female	PCS	1	1.17 (0.72-1.90)	-	-
CVD events by follow-up (y)	PCS				
< 10	PCS	6	1.27 (1.02-1.57)	78.9%	0.001
≥ 10	PCS	4	1.25 (1.01-1.56)	86.2%	0.001
CVD mortality by follow-up (y)	PCS				
< 10	PCS	5	1.16 (0.92-1.46)	54.8%	0.065
≥ 10	PCS	9	1.52 (1.30-1.77)	38%	0.115
CVD event by quality (NOS)	PCS				
Moderate quality	PCS	10	1.26 (1.10-1.45)	82.5%	0.001
High quality	PCS	1	1.03 (0.28-3.79)	-	-
CVD mortality by quality (NOS)	PCS				
Moderate quality	PCS	9	1.33 (1.09-1.61)	68%	0.002
High quality	PCS	5	1.49 (1.19-1.87)	36.9%	0.175
CVD events by CVD history at baseline	PCS				
No	PCS	7	1.28 (1.07-1.53)	84.5%	0.001
Yes	PCS	4	1.22 (0.92-1.61)	77.1%	0.004
CVD mortality by CVD history at baseline	PCS				
No	PCS	6	1.17 (0.92-1.47)	77.3%	0.001
Yes	PCS	8	1.57 (1.36-1.81)	2.7%	0.409

*Note.* RCT: Randomized controlled trial;PCS: Prospective cohort study; RR: Risk ratio; CI: Confidence interval; CVD: Cardiovascular diseases; ROB2: Risk of bias 2; NOS: Newcastle-Ottawa scale.

 Meta-regression was performed according to age, gender, and follow-up period, showing that with increasing age, the incidence of CVDs (R2 = 100%; b = - 0.008; standard error = 0.003; *P* = 0.04) and CVD mortality (R2 = 100%; b = - 0.014; standard error = 0.006; *P* = 0.04) decreases, as depicted in Tables S1 and S2.

###  Primary endpoint: prospective cohort studies

**Figure 4:**
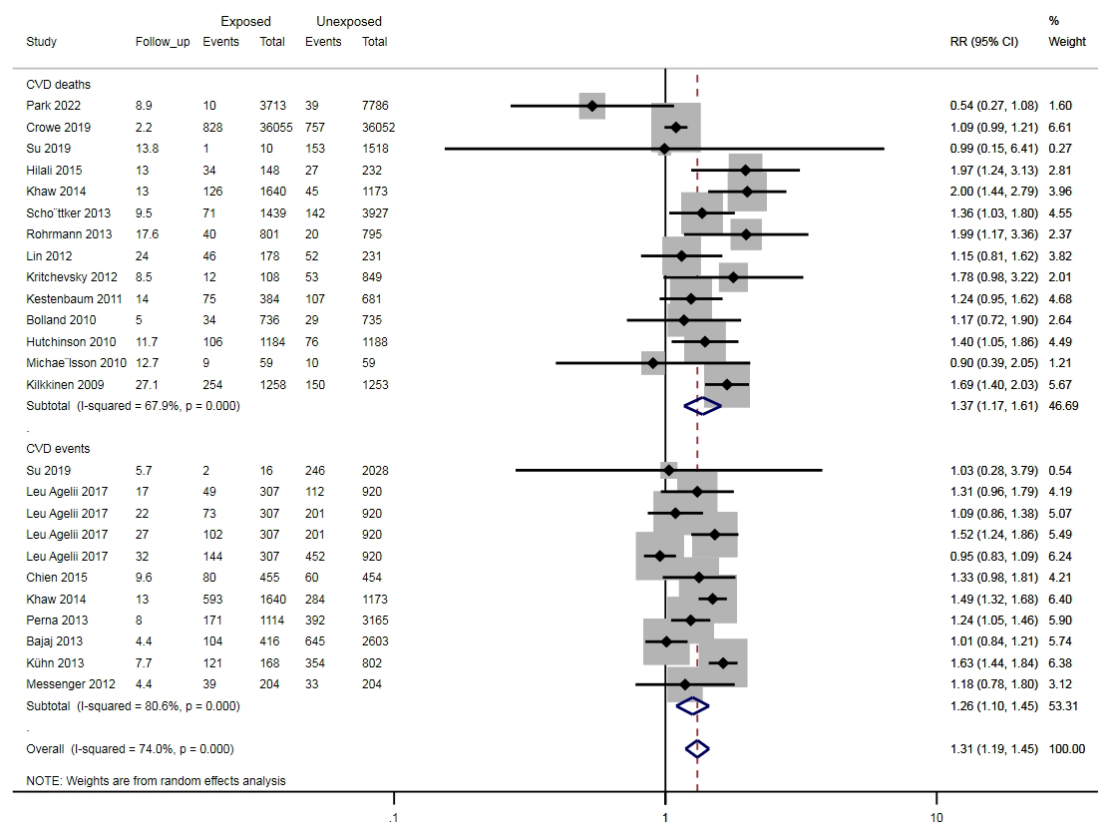
Forest plot for CVD events and mortality in PCSs. *Note.* CVD: Cardiovascular disease; PCSs: Prospective Cohort Studies

 The effects of vitamin D on CVDs were estimated using RR. RR (95% CI) for the highest vs. lowest categories of vitamin D was used in this study. In general, as Figure 4 indicates, circulating 25 (OH) D increased the risk of CVD incidence by 31% (RR: 1.44, 95% CI: 1.19-1.45) and CVD mortality by 37% (RR: 1.37, 95% CI: 1.17-1.61).

 The *P* value of Egger’s test for the CVD events was 0.55, and CVD mortality was 0.32. Sensitivity analysis for CVD events and mortality was performed by removing each study, which did not significantly change the general index of the study.

 Circulating 25 (OH) D increases the risk of MI and stroke by 47% (RR: 1.47, 95% CI: 1.17-1.86) and 42% (RR: 1.42, 95% CI: 1.18-1.70), respectively, as demonstrated in Figures S5 and S6. Further, subgroup analysis was conducted for the main outcome based on gender, follow-up period, study quality, and CVD history at baseline (Table 3). It was found that circulating 25 (OH) D increases the risk of CVDs by 28% in those without underlying CVDs (RR: 1.28, 95% CI: 1.07- 1.53), as shown in Table 3.

## Discussion

 This meta-analysis of cohort and clinical trials evaluated the effect of vitamin D on CVDs. The results showed that in PCSs, there is a direct association between vitamin D deficiency and the incidence of CVDs as well as its mortality, while in clinical trial studies, despite the inverse relationship between vitamin D and the incidence and mortality of CVDs, it was not statistically significant. Findings from this study suggested that as age rises, the risk of incidence and mortality of CVDs decreases.

 Despite relatively similar results from interventional studies regarding the relationship between vitamin D and subgroups of CVDs such as MI and stroke, which did not show significance, these results are consistent with findings from Barbarawi and colleagues’ study.^87^ In addition, our study’s results from prospective studies in subgroups such as MI, stroke, and CHD are relatively contradictory. As discussed in our study, most cohort studies support the association between vitamin D deficiency and enhanced risk of CVDs.

 Vitamin D receptors are found in most human cells and tissues, indicating many extraskeletal effects of this vitamin, especially in the cardiovascular system. Various mechanisms have been proposed in relation to vitamin D deficiency impacts on CVD risk factors such as the activation of the renin-angiotensin-aldosterone system, abnormal regulation of nitric oxide, oxidative stress, or changes in inflammatory pathways.^88^ The role of vitamin D has been attributed to the regulation of endothelial function. Moreover, endothelial dysfunction is strongly related to the pathogenesis of several cardiovascular disorders, atherosclerosis, and peripheral arterial diseases.^89^ Currently, there is no definitive agreement on the definition of optimal serum levels and nutritional requirements. In addition, the adequacy threshold may vary for different diseases and conditions, making it difficult to determine optimal reference values.^90^

 Observational studies showed that vitamin D deficiency is extremely common among people with CHD or HF and has a protective role in CVDs.^60,65^ In the Framingham Heart Study, low serum D 25 (OH) levels were associated with a 60% increase in cardiovascular death.^91^ A meta-analysis of several observational studies illustrated a positive relationship between low levels of vitamin D and the incidence of CVDs, HF, CHD, and mortality.^92^ However observational studies are susceptible to uncontrollable confounders by physical activity, nutritional status, and common chronic diseases that may affect serum vitamin D levels.^83^ According to the mentioned factors, we repeated subgroup analysis based on important confounding variables, but the role of residual confounding variables such as body mass index and physical activity cannot be fully controlled. Furthermore, a major confounding factor in observational studies could be the fact that people in good health may have higher 25 (OH) D levels due to more outdoor activity and, subsequently, more sun exposure.

 Subgroup analysis for interventional studies confirmed the overall results, but in cohort studies, although most of the results indicated a direct relationship between vitamin D deficiency and various CVDs in different subgroups, some of the results were contradictory. The incidence of CVDs in studies where the follow-up period of participants was less or more than 10 years revealed significant direct results. Previous studies have demonstrated a stronger relationship for follow-up periods of less than ten years, which may reflect greater changes in vitamin D over longer periods or competing risks for fatal and non-fatal diseases in older populations.^93^ Some studies have revealed a possible nonlinear relationship between vitamin D and CVD risk with a threshold effect or even a U-shaped relationship.^94,95^

 Findings from systematic review studies and meta-analyses of previous clinical trials confirmed the meta-analysis results of the present study.^87,96^ A large Mendelian randomization trial did not confirm the association between different levels of vitamin D and CVDs.^97^ In healthy and elderly subjects, daily supplementation with 4000 IU for one year did not significantly alter any of the cardiovascular risk factors, including arterial stiffness.^98^ In a double-blind, placebo-controlled trial in MI patients, daily administration of 4000 IU for five days affected some inflammatory indicators such as C-reactive protein and interleukin-6, while other indicators remained unchanged.^99^ In contrast, in the ViDA study, a monthly supplement of 100 000 international units of vitamin D over three years did not affect the incidence of CVDs, including atherosclerosis.^35^

 The Vitamin D Trial (VITAL) is a double-blind, randomized, placebo-controlled trial that investigated the effect of high-dose vitamin D (2000 IU) and omega-3 fatty acid supplementation in 25 871 participants. This study had a large and racially diverse general population sample, and the results of the study showed that the use of vitamin D supplementation does not lead to a significant difference in any of the CVDs compared to the placebo group.^18^ In addition, in the calcium-vitamin D trial for seven years, no reduction in the incidence of CHD or stroke was observed with the combination of calcium and vitamin D supplementation.^49^ Such differences may be the result of different doses and times. Overall, the results of recent RCTs clearly indicate that vitamin D supplementation in people with adequate levels of vitamin D is not significantly associated with CVDs in the general population.

 According to the results of our study regarding clinical trial studies, age increases the risk of incidence and death of CVDs, and the results of analysis of other studies according to age have demonstrated a significant relationship between increasing age and the incidence of CVDs.^87,96^ Nevertheless, this relationship was not significant in terms of gender, excess calcium consumption (less than 25 ng/mL and more), body mass index, vitamin D dose, and other factors. The regression analysis results for age in Barbarawi and colleagues’ study showed that it should be interpreted cautiously in the presence of other variables.^87^

 The present study is the first one that includes two designs, namely, RCTs and PCSs with a large sample size, considering the number of included articles. This study also had some limitations. First, most clinical trial studies were not designed to evaluate the effects of vitamin D supplementation on CVDs, yet their primary outcome was the effect of vitamin D on fractures and osteoporosis in elderly and postmenopausal women, and CVDs were considered secondary outcomes and were underpowered for CVD events. Second, some studies did not have enough data to calculate the effect of the study (RR). Third, it was impossible to access some articles’ full text.

**Highlights**

- Among 134 384 participants entering the clinical trials, 67 665 were taking vitamin D, and 66 719 were not taking vitamin D.
- In clinical trials, 17 studies (58.6%) were classified as low risk, four studies (13.8%) as some concerns, and eight studies (27.6%) as high risk.
- In clinical trial studies, the incidence of CVDs among the vitamin D-consuming group was not significantly different from that of the placebo group (RR: 0.99, 95% CI: 0.95-1.03; *P* = 0.770; I^2^ = 0%).
- Circulating 25 (OH) D increases the risk of MI and stroke by 47% (RR: 1.47, 95% CI: 1.17-1.86) and 42% (RR: 1.42, 95% CI: 1.18-1.70), respectively.

## Conclusion

 According to the results of the current study regarding clinical trial studies, age increases the risk of incidence and death of CVDs. According to the findings of systematic reviews and meta-analyses of RCTs, it appears that vitamin D supplementation may have a small overall survival benefit. However, there is a direct association between vitamin D deficiency and the incidence of CVDs as well as its mortality. According to the results of clinical trial studies, which carry higher levels of scientific evidence, it can be concluded that vitamin D supplementation does not exert a significant effect on the incidence, mortality, and reduction of CVDs.

## Acknowledgments

 The authors appreciate the collaboration of the Clinical Research Development Unit, Kowsar Hospital, Sanandaj, Iran.

## Authors’ Contribution

**Conceptualization:** Fatemeh Gholami, Ghobad Moradi, Ako Mahmoudian.

**Data curation:** Fatemeh Gholami, Ghobad Moradi, Mohammad Aziz Rasouli.

**Formal analysis:** Fatemeh Gholami.

**Investigation:** Fatemeh Gholami, Mohammad Aziz Rasouli.

**Methodology:** Fatemeh Gholami, Ghobad Moradi, Mohammad Aziz Rasouli.

**Project administration:** Fatemeh Gholami, Ghobad Moradi.

**Resources:** Shahram Darvishzadehdaledari, Zeynab Alizadeh, Fatemeh Gholami.

**Software:** Fatemeh Gholami.

**Supervision:** Ghobad Moradi.

**Validation:** Ghobad Moradi, Ako Mahmoudian.

**Visualization:** Fatemeh Gholami, Mohammad Aziz Rasouli.

**Writing–original draft:** Fatemeh Gholami, Mohammad Aziz Rasouli, Zeynab Alizadeh, Shahram Darvishzadehdaledari.

**Writing–review & editing:** Fatemeh Gholami, Mohammad Aziz Rasouli.

## Competing Interests

 No competing interests.

## Ethical Approval

 Not applicable.

## Funding

 There was no funding.

## Supplementary Files

Supplementary file 1 contains Tables S1-S2 and Figures S1-S6.

## References

[1] Mensah GA, Wei GS, Sorlie PD, Fine LJ, Rosenberg Y, Kaufmann PG (2017). Decline in cardiovascular mortality: possible causes and implications. Circ Res.

[2] Curry SJ, Krist AH, Owens DK, Barry MJ, Caughey AB, Davidson KW (2018). Risk assessment for cardiovascular disease with nontraditional risk factors: US preventive services task force recommendation statement. JAMA.

[3] Roth GA, Johnson C, Abajobir A, Abd-Allah F, Abera SF, Abyu G (2017). Global, regional, and national burden of cardiovascular diseases for 10 causes, 1990 to 2015. J Am Coll Cardiol.

[4] Judd SE, Tangpricha V (2009). Vitamin D deficiency and risk for cardiovascular disease. Am J Med Sci.

[5] Bays HE, Kulkarni A, German C, Satish P, Iluyomade A, Dudum R (2022). Ten things to know about ten cardiovascular disease risk factors - 2022. Am J Prev Cardiol.

[6] Norman PE, Powell JT (2014). Vitamin D and cardiovascular disease. Circ Res.

[7] Amrein K, Scherkl M, Hoffmann M, Neuwersch-Sommeregger S, Köstenberger M, Tmava Berisha A (2020). Vitamin D deficiency 20: an update on the current status worldwide. Eur J Clin Nutr.

[8] Palacios C, Gonzalez L (2014). Is vitamin D deficiency a major global public health problem?. J Steroid Biochem Mol Biol.

[9] Mithal A, Wahl DA, Bonjour JP, Burckhardt P, Dawson-Hughes B, Eisman JA (2009). Global vitamin D status and determinants of hypovitaminosis D. Osteoporos Int.

[10] Lee JH, O’Keefe JH, Bell D, Hensrud DD, Holick MF (2008). Vitamin D deficiency an important, common, and easily treatable cardiovascular risk factor?. J Am Coll Cardiol.

[11] Haq A, Svobodová J, Imran S, Stanford C, Razzaque MS (2016). Vitamin D deficiency: a single centre analysis of patients from 136 countries. J Steroid Biochem Mol Biol.

[12] Dziedzic EA, Gąsior JS, Pawłowski M, Wodejko-Kucharska B, Saniewski T, Marcisz A (2019). Vitamin D level is associated with severity of coronary artery atherosclerosis and incidence of acute coronary syndromes in non-diabetic cardiac patients. Arch Med Sci.

[13] Dziedzic EA, Przychodzeń S, Dąbrowski M (2016). The effects of vitamin D on severity of coronary artery atherosclerosis and lipid profile of cardiac patients. Arch Med Sci.

[14] Institute of Medicine (US) Standing Committee on the Scientific Evaluation of Dietary Reference Intakes. Dietary Reference Intakes for Calcium, Phosphorus, Magnesium, Vitamin D, and Fluoride. Washington, DC: National Academies Press (US); 1997. 23115811

[15] Ministerråd N. Nordic Nutrition Recommendations 2012. Part 1: Summary, Principles and Use. Nordic Council of Ministers; 2013.

[16] Zittermann A, Trummer C, Theiler-Schwetz V, Lerchbaum E, März W, Pilz S (2021). Vitamin D and cardiovascular disease: an updated narrative review. Int J Mol Sci.

[17] Zittermann A (2018). Vitamin D status, supplementation and cardiovascular disease. Anticancer Res.

[18] Manson JE, Cook NR, Lee IM, Christen W, Bassuk SS, Mora S (2019). Vitamin D supplements and prevention of cancer and cardiovascular disease. N Engl J Med.

[19] Mez J, Daneshvar DH, Kiernan PT, Abdolmohammadi B, Alvarez VE, Huber BR (2017). Clinicopathological evaluation of chronic traumatic encephalopathy in players of American football. JAMA.

[20] Rohrmann S, Braun J, Bopp M, Faeh D (2013). Inverse association between circulating vitamin D and mortality--dependent on sex and cause of death?. Nutr Metab Cardiovasc Dis.

[21] Park D, Lee J, Park CY, Shin MJ (2022). Low vitamin D status is associated with increased risk of mortality in Korean men and adults with hypertension: a population-based cohort study. Nutrients.

[22] Moher D, Liberati A, Tetzlaff J, Altman DG (2009). Preferred reporting items for systematic reviews and meta-analyses: the PRISMA statement. PLoS Med.

[23] Wells G, Shea B, O’Connell D, Peterson J, Welch V, Losos M, et al. Newcastle-Ottawa Quality Assessment Scale Cohort Studies. University of Ottawa; 2014.

[24] Lo CK, Mertz D, Loeb M (2014). Newcastle-Ottawa Scale: comparing reviewers’ to authors’ assessments. BMC Med Res Methodol.

[25] Higgins JP, Savović J, Page MJ, Elbers RG, Sterne JA. Assessing risk of bias in a randomized trial. In: Cochrane Handbook for Systematic Reviews of Interventions. John Wiley & Sons; 2019. p. 205-28.

[26] Sterne JA, Egger M. Regression methods to detect publication and other bias in meta‐analysis. In: Publication Bias in Meta‐Analysis: Prevention, Assessment and Adjustments. John Wiley & Sons; 2005. p. 99-110.

[27] gger M, Davey Smith G, Schneider M, Minder C (1997). Bias in meta-analysis detected by a simple, graphical test. BMJ.

[28] Higgins JP, Thompson SG (2002). Quantifying heterogeneity in a meta-analysis. Stat Med.

[29] Tobias A (1999). Assessing the influence of a single study in the meta-analysis estimate. Stata Tech Bull.

[30] Thorlund K, Devereaux PJ, Wetterslev J, Guyatt G, Ioannidis JP, Thabane L (2009). Can trial sequential monitoring boundaries reduce spurious inferences from meta-analyses?. Int J Epidemiol.

[31] Virtanen JK, Nurmi T, Aro A, Bertone-Johnson ER, Hyppönen E, Kröger H (2022). Vitamin D supplementation and prevention of cardiovascular disease and cancer in the Finnish Vitamin D Trial: a randomized controlled trial. Am J Clin Nutr.

[32] Neale RE, Baxter C, Romero BD, McLeod DSA, English DR, Armstrong BK (2022). The D-Health Trial: a randomised controlled trial of the effect of vitamin D on mortality. Lancet Diabetes Endocrinol.

[33] Chatterjee R, Fuss P, Vickery EM, LeBlanc ES, Sheehan PR, Lewis MR (2021). Vitamin D supplementation for prevention of cancer: the D2d cancer outcomes (D2dCA) ancillary study. J Clin Endocrinol Metab.

[34] Shoji T, Inaba M, Fukagawa M, Ando R, Emoto M, Fujii H (2018). Effect of oral alfacalcidol on clinical outcomes in patients without secondary hyperparathyroidism receiving maintenance hemodialysis: the J-DAVID randomized clinical trial. JAMA.

[35] Scragg R, Stewart AW, Waayer D, Lawes CMM, Toop L, Sluyter J (2017). Effect of monthly high-dose vitamin D supplementation on cardiovascular disease in the vitamin D assessment study: a randomized clinical trial. JAMA Cardiol.

[36] Zittermann A, Ernst JB, Prokop S, Fuchs U, Dreier J, Kuhn J (2017). Effect of vitamin D on all-cause mortality in heart failure (EVITA): a 3-year randomized clinical trial with 4000 IU vitamin D daily. Eur Heart J.

[37] Jorde R, Sollid ST, Svartberg J, Schirmer H, Joakimsen RM, Njølstad I (2016). Vitamin D 20,000 IU per week for five years does not prevent progression from prediabetes to diabetes. J Clin Endocrinol Metab.

[38] Baron JA, Barry EL, Mott LA, Rees JR, Sandler RS, Snover DC (2015). A trial of calcium and vitamin D for the prevention of colorectal adenomas. N Engl J Med.

[39] Martineau AR, MacLaughlin BD, Hooper RL, Barnes NC, Jolliffe DA, Greiller CL (2015). Double-blind randomised placebo-controlled trial of bolus-dose vitamin D3 supplementation in adults with asthma (ViDiAs). Thorax.

[40] Ford JA, MacLennan GS, Avenell A, Bolland M, Grey A, Witham M (2014). Cardiovascular disease and vitamin D supplementation: trial analysis, systematic review, and meta-analysis. Am J Clin Nutr.

[41] Wang AY, Fang F, Chan J, Wen YY, Qing S, Chan IH (2014). Effect of paricalcitol on left ventricular mass and function in CKD--the OPERA trial. J Am Soc Nephrol.

[42] Witham MD, Price RJ, Struthers AD, Donnan PT, Messow CM, Ford I (2013). Cholecalciferol treatment to reduce blood pressure in older patients with isolated systolic hypertension: the VitDISH randomized controlled trial. JAMA Intern Med.

[43] Gallagher JC, Sai A, Templin T 2nd, Smith L (2012). Dose response to vitamin D supplementation in postmenopausal women: a randomized trial. Ann Intern Med.

[44] Lehouck A, Mathieu C, Carremans C, Baeke F, Verhaegen J, Van Eldere J (2012). High doses of vitamin D to reduce exacerbations in chronic obstructive pulmonary disease: a randomized trial. Ann Intern Med.

[45] Sanders KM, Stuart AL, Williamson EJ, Simpson JA, Kotowicz MA, Young D (2010). Annual high-dose oral vitamin D and falls and fractures in older women: a randomized controlled trial. JAMA.

[46] Prince RL, Austin N, Devine A, Dick IM, Bruce D, Zhu K (2008). Effects of ergocalciferol added to calcium on the risk of falls in elderly high-risk women. Arch Intern Med.

[47] Zhu K, Devine A, Dick IM, Wilson SG, Prince RL (2008). Effects of calcium and vitamin D supplementation on hip bone mineral density and calcium-related analytes in elderly ambulatory Australian women: a five-year randomized controlled trial. J Clin Endocrinol Metab.

[48] Berggren M, Stenvall M, Olofsson B, Gustafson Y (2008). Evaluation of a fall-prevention program in older people after femoral neck fracture: a one-year follow-up. Osteoporos Int.

[49] Hsia J, Heiss G, Ren H, Allison M, Dolan NC, Greenland P (2007). Calcium/vitamin D supplementation and cardiovascular events. Circulation.

[50] Jackson RD, LaCroix AZ, Gass M, Wallace RB, Robbins J, Lewis CE (2006). Calcium plus vitamin D supplementation and the risk of fractures. N Engl J Med.

[51] Brazier M, Grados F, Kamel S, Mathieu M, Morel A, Maamer M (2005). Clinical and laboratory safety of one year’s use of a combination calcium + vitamin D tablet in ambulatory elderly women with vitamin D insufficiency: results of a multicenter, randomized, double-blind, placebo-controlled study. Clin Ther.

[52] Grant AM, Avenell A, Campbell MK, McDonald AM, MacLennan GS, McPherson GC (2005). Oral vitamin D3 and calcium for secondary prevention of low-trauma fractures in elderly people (Randomised Evaluation of Calcium Or vitamin D, RECORD): a randomised placebo-controlled trial. Lancet.

[53] Trivedi DP, Doll R, Khaw KT (2003). Effect of four monthly oral vitamin D3 (cholecalciferol) supplementation on fractures and mortality in men and women living in the community: randomised double blind controlled trial. BMJ.

[54] Komulainen M, Kröger H, Tuppurainen MT, Heikkinen AM, Alhava E, Honkanen R (1999). Prevention of femoral and lumbar bone loss with hormone replacement therapy and vitamin D3 in early postmenopausal women: a population-based 5-year randomized trial. J Clin Endocrinol Metab.

[55] Ott SM, Chesnut CH 3rd (1989). Calcitriol treatment is not effective in postmenopausal osteoporosis. Ann Intern Med.

[56] Aloia JF, Vaswani A, Yeh JK, Ellis K, Yasumura S, Cohn SH (1988). Calcitriol in the treatment of postmenopausal osteoporosis. Am J Med.

[57] Inkovaara J, Gothoni G, Halttula R, Heikinheimo R, Tokola O (1983). Calcium, vitamin D and anabolic steroid in treatment of aged bones: double-blind placebo-controlled long-term clinical trial. Age Ageing.

[58] Brohult J, Jonson B (1973). Effects of large doses of calciferol on patients with rheumatoid arthritis A double-blind clinical trial. Scand J Rheumatol.

[59] Heath AK, Hodge AM, Ebeling PR, Kvaskoff D, Eyles DW, Giles GG (2020). Circulating 25-hydroxyvitamin D concentration and cause-specific mortality in the Melbourne Collaborative Cohort Study. J Steroid Biochem Mol Biol.

[60] Paul S, Judd SE, Howard VJ, Safford MS, Gutiérrez OM (2019). Association of 25-hydroxyvitamin D with incident coronary heart disease in the Reasons for Geographic and Racial Differences in Stroke (REGARDS) study. Am Heart J.

[61] Crowe FL, Thayakaran R, Gittoes N, Hewison M, Thomas GN, Scragg R (2019). Non-linear associations of 25-hydroxyvitamin D concentrations with risk of cardiovascular disease and all-cause mortality: results from The Health Improvement Network (THIN) database. J Steroid Biochem Mol Biol.

[62] Su Y, Leung J, Lee J, Ho KF, Kwok T (2020). The effect of physical activity on dose-relationship between serum 25-hydroxyvitamin D and cardiovascular health events in older adults. Nutr Metab Cardiovasc Dis.

[63] Leu Agelii M, Lehtinen-Jacks S, Zetterberg H, Sundh V, Björkelund C, Lissner L (2017). Low vitamin D status in relation to cardiovascular disease and mortality in Swedish women - effect of extended follow-up. Nutr Metab Cardiovasc Dis.

[64] El Hilali J, de Koning EJ, van Ballegooijen AJ, Lips P, Sohl E, van Marwijk HWJ (2016). Vitamin D, PTH and the risk of overall and disease-specific mortality: results of the Longitudinal Aging Study Amsterdam. J Steroid Biochem Mol Biol.

[65] Lutsey PL, Michos ED, Misialek JR, Pankow JS, Loehr L, Selvin E (2015). Race and vitamin D binding protein gene polymorphisms modify the association of 25-hydroxyvitamin D and incident heart failure: the ARIC (Atherosclerosis Risk in Communities) study. JACC Heart Fail.

[66] Chien KL, Hsu HC, Chen PC, Lin HJ, Su TC, Chen MF (2015). Total 25-hydroxyvitamin D concentration as a predictor for all-cause death and cardiovascular event risk among ethnic Chinese adults: a cohort study in a Taiwan community. PLoS One.

[67] Michos ED, Misialek JR, Selvin E, Folsom AR, Pankow JS, Post WS (2015). 25-hydroxyvitamin D levels, vitamin D binding protein gene polymorphisms and incident coronary heart disease among whites and blacks: the ARIC study. Atherosclerosis.

[68] Khaw KT, Luben R, Wareham N (2014). Serum 25-hydroxyvitamin D, mortality, and incident cardiovascular disease, respiratory disease, cancers, and fractures: a 13-y prospective population study. Am J Clin Nutr.

[69] Wannamethee SG, Welsh P, Papacosta O, Lennon L, Whincup PH, Sattar N (2014). Elevated parathyroid hormone, but not vitamin D deficiency, is associated with increased risk of heart failure in older men with and without cardiovascular disease. Circ Heart Fail.

[70] Perna L, Schöttker B, Holleczek B, Brenner H (2013). Serum 25-hydroxyvitamin D and incidence of fatal and nonfatal cardiovascular events: a prospective study with repeated measurements. J Clin Endocrinol Metab.

[71] Bajaj A, Stone KL, Peters K, Parimi N, Barrett-Connor E, Bauer D (2014). Circulating vitamin D, supplement use, and cardiovascular disease risk: the MrOS Sleep Study. J Clin Endocrinol Metab.

[72] Schöttker B, Haug U, Schomburg L, Köhrle J, Perna L, Müller H (2013). Strong associations of 25-hydroxyvitamin D concentrations with all-cause, cardiovascular, cancer, and respiratory disease mortality in a large cohort study. Am J Clin Nutr.

[73] Kühn T, Kaaks R, Teucher B, Hirche F, Dierkes J, Weikert C (2013). Plasma 25-hydroxyvitamin D and its genetic determinants in relation to incident myocardial infarction and stroke in the European prospective investigation into cancer and nutrition (EPIC)-Germany study. PLoS One.

[74] Robinson-Cohen C, Hoofnagle AN, Ix JH, Sachs MC, Tracy RP, Siscovick DS (2013). Racial differences in the association of serum 25-hydroxyvitamin D concentration with coronary heart disease events. JAMA.

[75] Schierbeck LL, Rejnmark L, Tofteng CL, Stilgren L, Eiken P, Mosekilde L (2012). Vitamin D deficiency in postmenopausal, healthy women predicts increased cardiovascular events: a 16-year follow-up study. Eur J Endocrinol.

[76] Lin SW, Chen W, Fan JH, Dawsey SM, Taylor PR, Qiao YL (2012). Prospective study of serum 25-hydroxyvitamin D concentration and mortality in a Chinese population. Am J Epidemiol.

[77] Kritchevsky SB, Tooze JA, Neiberg RH, Schwartz GG, Hausman DB, Johnson MA (2012). 25-Hydroxyvitamin D, parathyroid hormone, and mortality in black and white older adults: the health ABC study. J Clin Endocrinol Metab.

[78] Messenger W, Nielson CM, Li H, Beer T, Barrett-Connor E, Stone K (2012). Serum and dietary vitamin D and cardiovascular disease risk in elderly men: a prospective cohort study. Nutr Metab Cardiovasc Dis.

[79] Kestenbaum B, Katz R, de Boer I, Hoofnagle A, Sarnak MJ, Shlipak MG (2011). Vitamin D, parathyroid hormone, and cardiovascular events among older adults. J Am Coll Cardiol.

[80] Bansal N, Zelnick L, Robinson-Cohen C, Hoofnagle AN, Ix JH, Lima JA (2014). Serum parathyroid hormone and 25-hydroxyvitamin D concentrations and risk of incident heart failure: the Multi-Ethnic Study of Atherosclerosis. J Am Heart Assoc.

[81] Bolland MJ, Bacon CJ, Horne AM, Mason BH, Ames RW, Wang TK (2010). Vitamin D insufficiency and health outcomes over 5 y in older women. Am J Clin Nutr.

[82] Hutchinson MS, Grimnes G, Joakimsen RM, Figenschau Y, Jorde R (2010). Low serum 25-hydroxyvitamin D levels are associated with increased all-cause mortality risk in a general population: the Tromsø study. Eur J Endocrinol.

[83] Michaëlsson K, Baron JA, Snellman G, Gedeborg R, Byberg L, Sundström J (2010). Plasma vitamin D and mortality in older men: a community-based prospective cohort study. Am J Clin Nutr.

[84] Kilkkinen A, Knekt P, Aro A, Rissanen H, Marniemi J, Heliövaara M (2009). Vitamin D status and the risk of cardiovascular disease death. Am J Epidemiol.

[85] Giovannucci E, Liu Y, Hollis BW, Rimm EB (2008). 25-hydroxyvitamin D and risk of myocardial infarction in men: a prospective study. Arch Intern Med.

[86] Pilz S, März W, Wellnitz B, Seelhorst U, Fahrleitner-Pammer A, Dimai HP (2008). Association of vitamin D deficiency with heart failure and sudden cardiac death in a large cross-sectional study of patients referred for coronary angiography. J Clin Endocrinol Metab.

[87] Barbarawi M, Kheiri B, Zayed Y, Barbarawi O, Dhillon H, Swaid B (2019). Vitamin D supplementation and cardiovascular disease risks in more than 83 000 individuals in 21 randomized clinical trials: a meta-analysis. JAMA Cardiol.

[88] de la Guía-Galipienso F, Martínez-Ferran M, Vallecillo N, Lavie CJ, Sanchis-Gomar F, Pareja-Galeano H (2021). Vitamin D and cardiovascular health. Clin Nutr.

[89] Legarth C, Grimm D, Wehland M, Bauer J, Krüger M (2018). The impact of vitamin D in the treatment of essential hypertension. Int J Mol Sci.

[90] Latic N, Erben RG (2020). Vitamin D and cardiovascular disease, with emphasis on hypertension, atherosclerosis, and heart failure. Int J Mol Sci.

[91] Mahmood SS, Levy D, Vasan RS, Wang TJ (2014). The Framingham Heart Study and the epidemiology of cardiovascular disease: a historical perspective. Lancet.

[92] Brøndum-Jacobsen P, Benn M, Jensen GB, Nordestgaard BG (2012). 25-hydroxyvitamin d levels and risk of ischemic heart disease, myocardial infarction, and early death: population-based study and meta-analyses of 18 and 17 studies. Arterioscler Thromb Vasc Biol.

[93] Wang L, Song Y, Manson JE, Pilz S, März W, Michaëlsson K (2012). Circulating 25-hydroxy-vitamin D and risk of cardiovascular disease: a meta-analysis of prospective studies. Circ Cardiovasc Qual Outcomes.

[94] Wang TJ, Pencina MJ, Booth SL, Jacques PF, Ingelsson E, Lanier K (2008). Vitamin D deficiency and risk of cardiovascular disease. Circulation.

[95] Melamed ML, Michos ED, Post W, Astor B (2008). 25-hydroxyvitamin D levels and the risk of mortality in the general population. Arch Intern Med.

[96] Pei YY, Zhang Y, Peng XC, Liu ZR, Xu P, Fang F (2022). Association of vitamin D supplementation with cardiovascular events: a systematic review and meta-analysis. Nutrients.

[97] Manousaki D, Mokry LE, Ross S, Goltzman D, Richards JB (2016). Mendelian randomization studies do not support a role for vitamin D in coronary artery disease. Circ Cardiovasc Genet.

[98] Hin H, Tomson J, Newman C, Kurien R, Lay M, Cox J (2017). Optimum dose of vitamin D for disease prevention in older people: BEST-D trial of vitamin D in primary care. Osteoporos Int.

[99] Sokol SI, Srinivas V, Crandall JP, Kim M, Tellides G, Lebastchi AH (2012). The effects of vitamin D repletion on endothelial function and inflammation in patients with coronary artery disease. Vasc Med.

