# Supplementary material for: Vitamin D Supplementation and Cardiovascular Disease Risks in More Than 134000 Individuals in 29 Randomized Clinical Trials and 157000 Individuals in 30 Prospective Cohort Studies: An Updated Systematic Review and Meta-analysis
Source: J Res Health Sci. 2023 Dec 29;23(4):e00594. doi: 10.34172/jrhs.2023.129 (PMC10843321; doi:10.34172/jrhs.2023.129)
Supplement: Supplementary file 1 — contains Tables S1-S2 and Figures S1-S6. [file jrhs-23-e00594-s001.pdf]

JRHS 2023; 23(4):e00594

doi:10.34172/jrhs.2023.129

Table S1: Meta-regression for CVD events by age in Randomized controlled trial

LnRR	Coefficient	SE	t	P-value	95% CI	
					Lower limit	Upper limit
age	-0.008	0.003	-2.28	0.036	-0.015	-0.0006
constant	0.522	0.233	2.24	0.039	0.028	1.015
Number of observations=18; Adjusted R^2=100%						

Table S2: Meta-regression for CVD deaths by age in Randomized controlled trial

LnRR	Coefficient	SE	t	P-value	95% CI	
					Lower limit	Upper limit
age	-0.014	0.006	-2.30	0.036	-0.027	-0.001
constant	0.921	0.414	2.22	0.042	0.038	1.804
Number of observations=17; Adjusted R^2=100%						

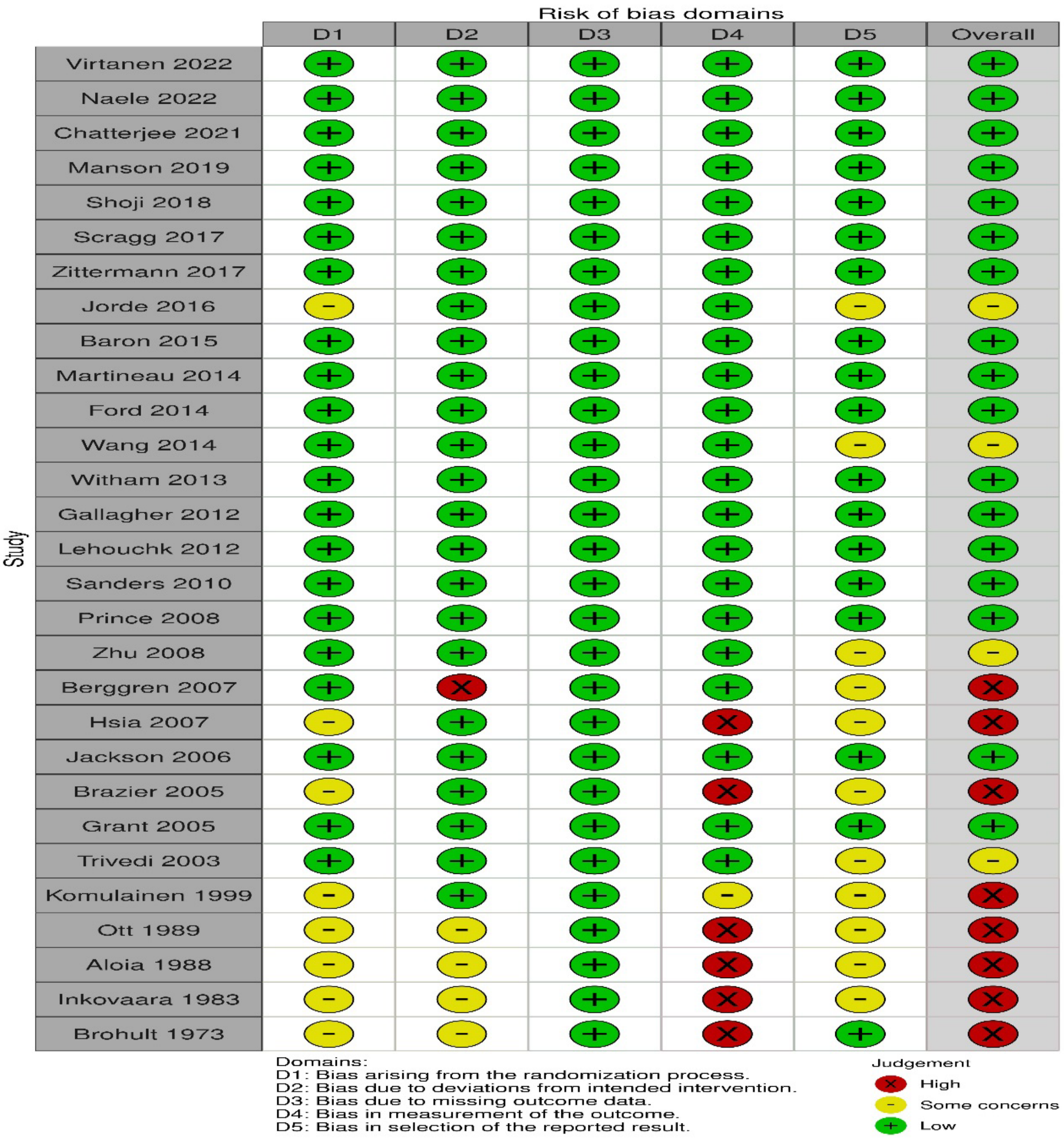

Figure S1: Risk of bias in Randomized Controlled Trial

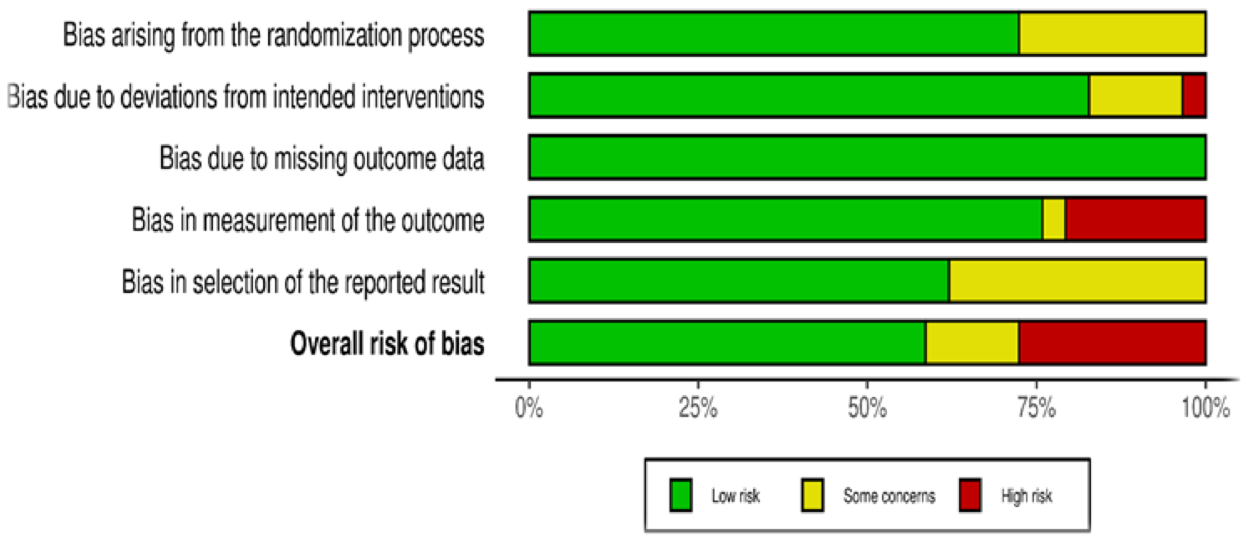

Figure S2: Risk of Bias in Randomized Controlled Trials

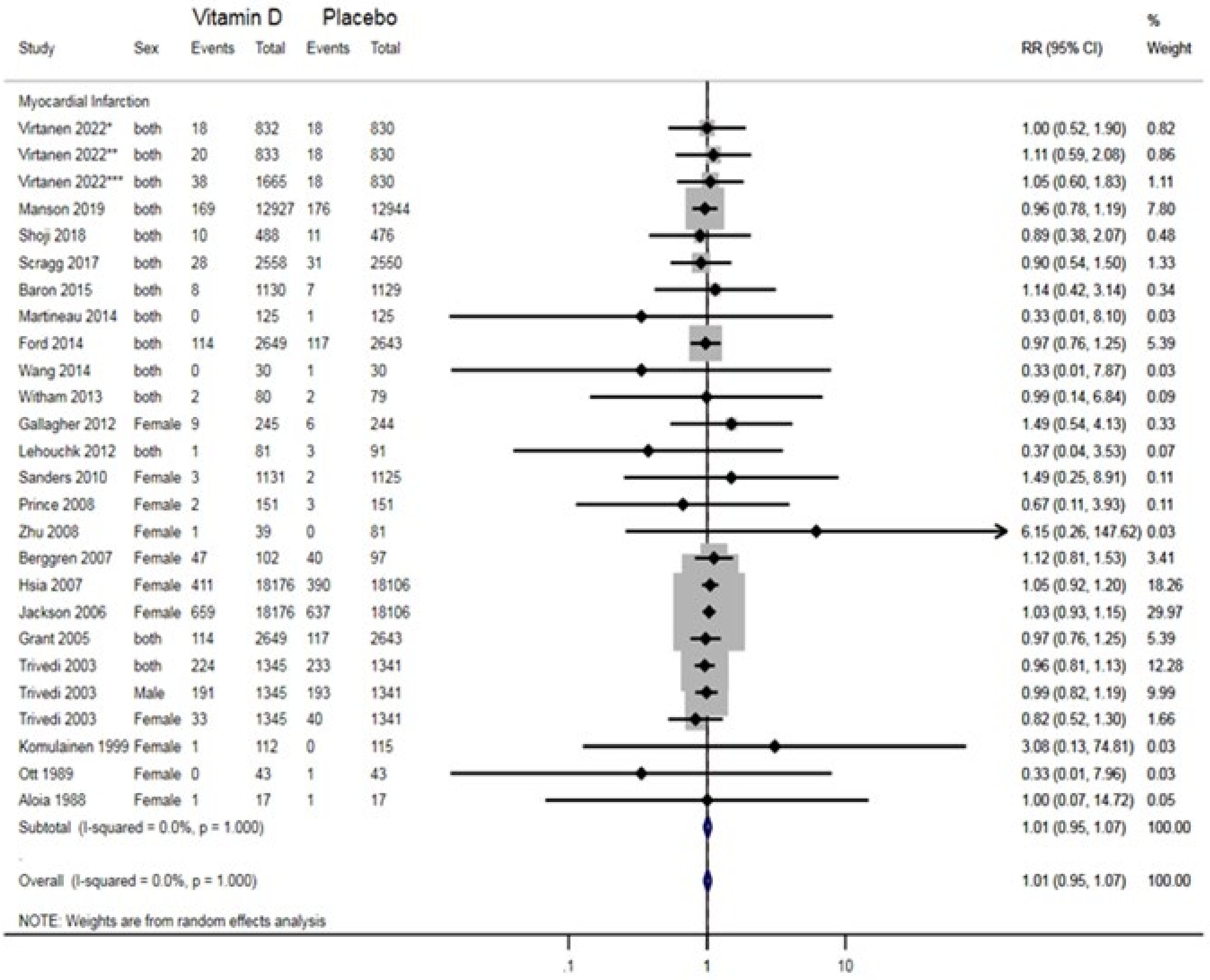

Figure S3: Forrest plot for Myocardial Infarction in Randomized Controlled Trials

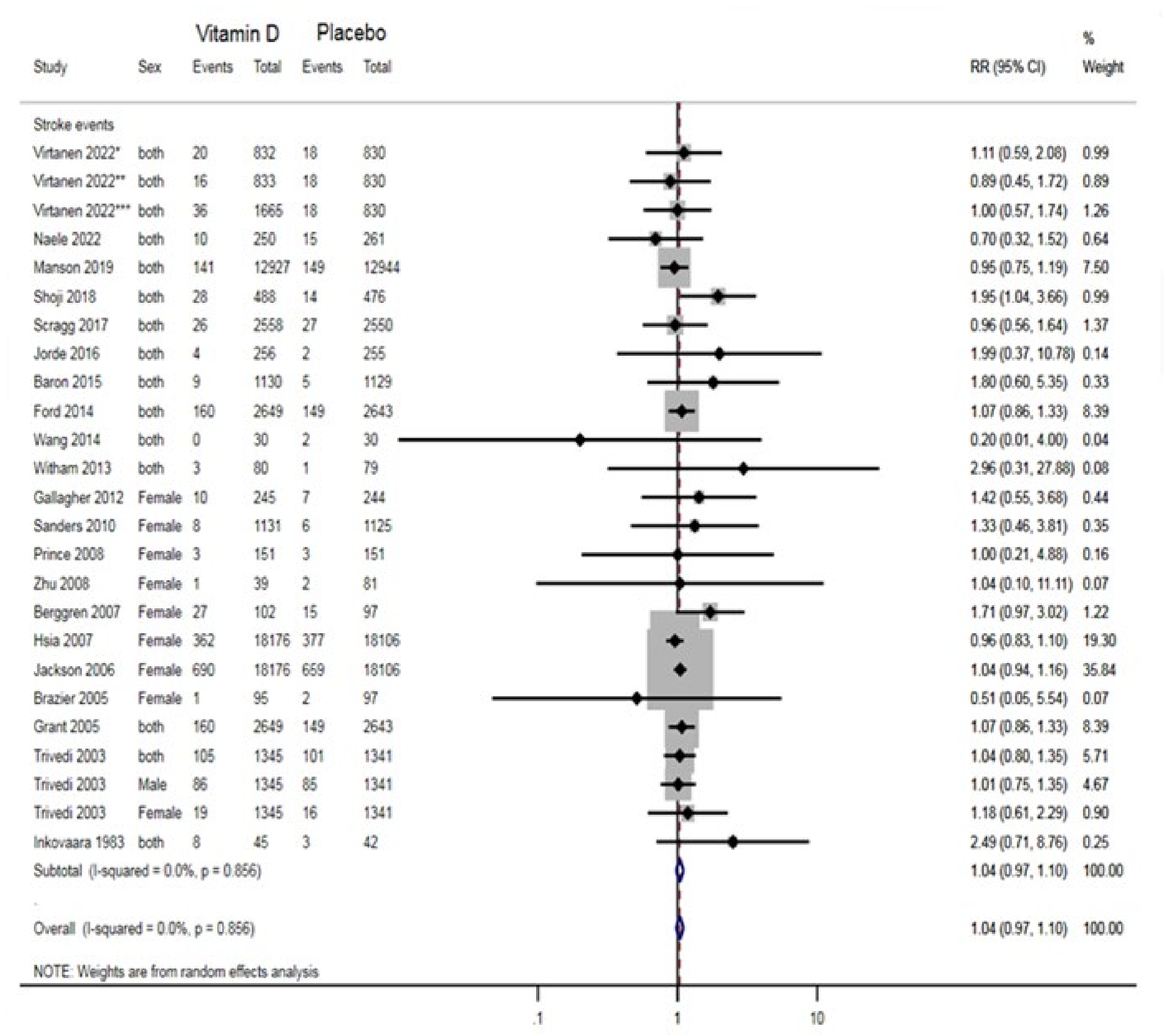

Figure S4: Forrest plot for Stroke in Randomized Controlled Trials

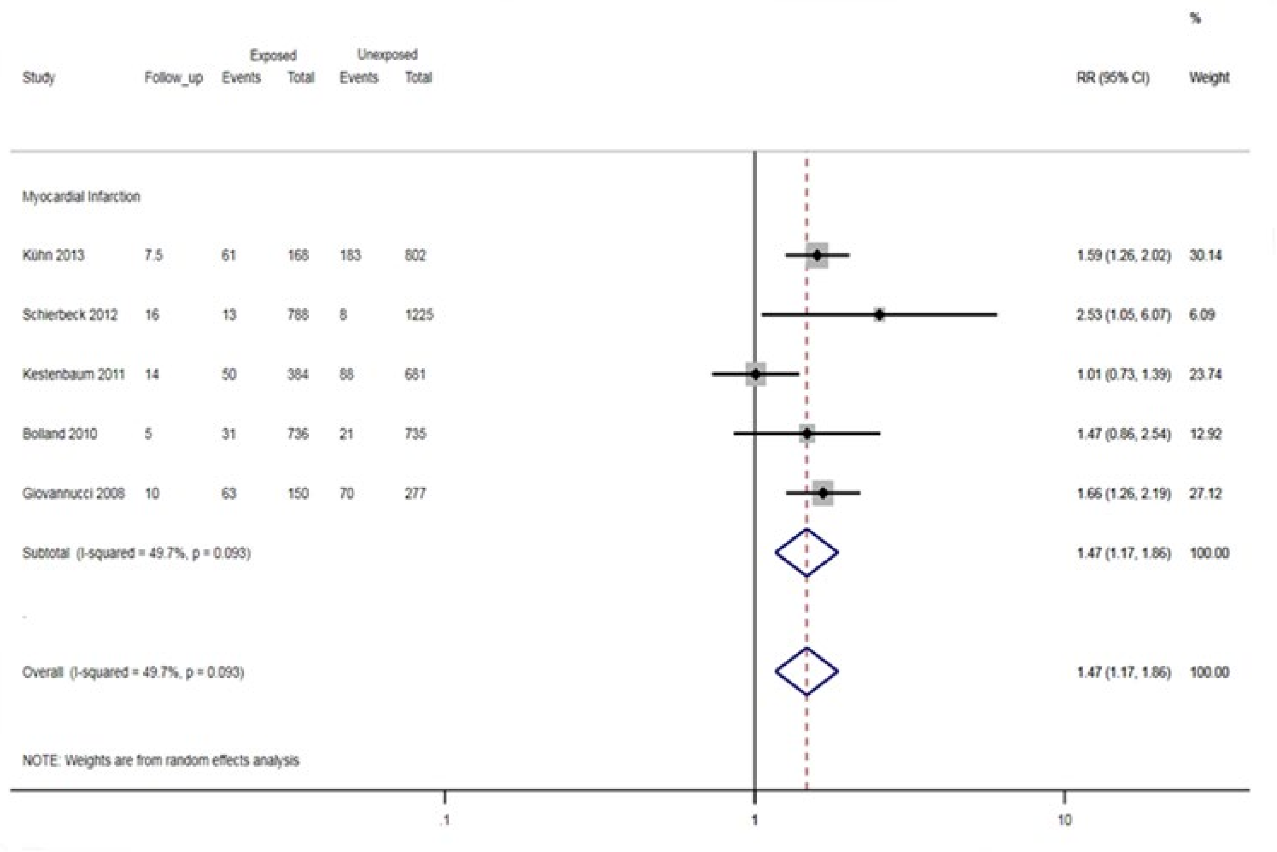

Figure S5: Forest plot for Myocardial Infarction in Prospective Cohort Studies

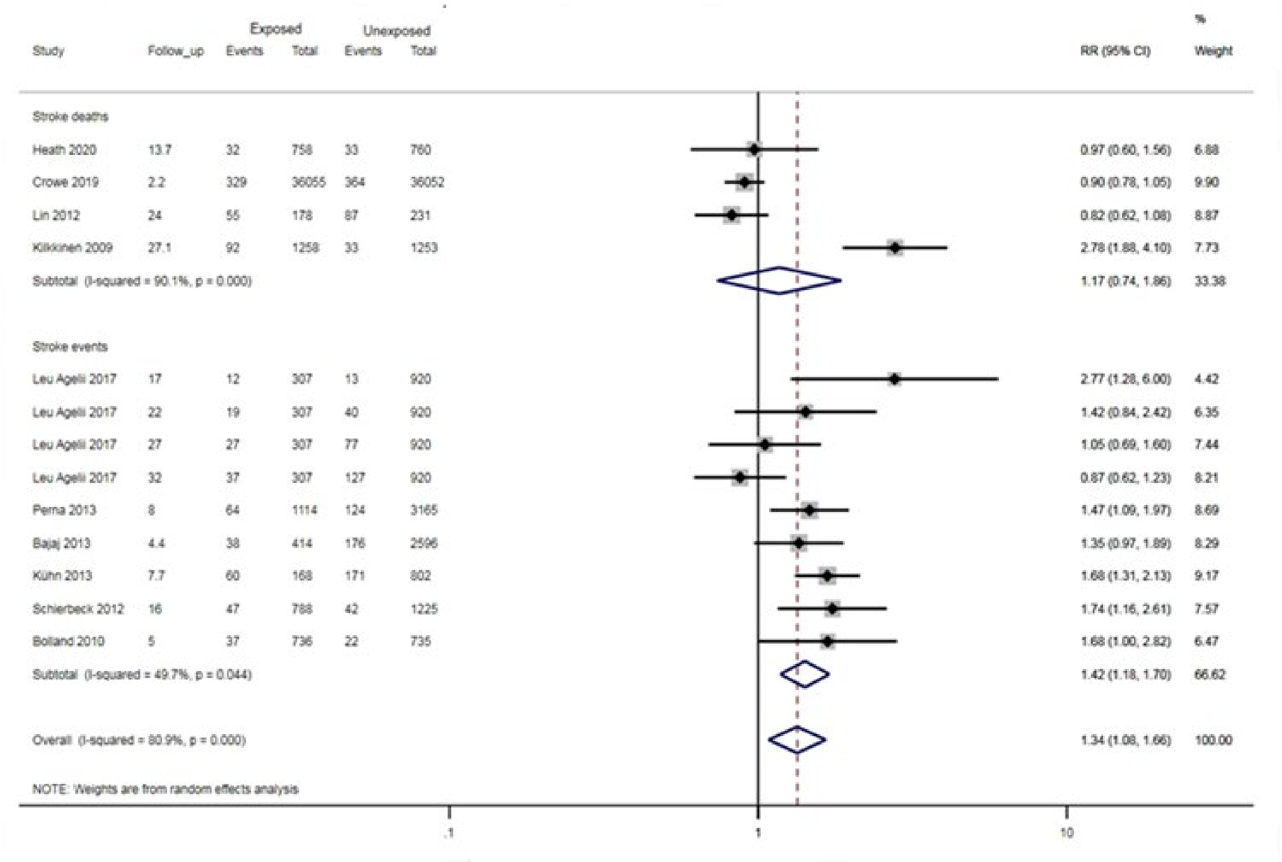

Figure S6: Forest plot for Stroke in Prospective Cohort Studies
